# Uterine NDRG2 expression is increased at implantation sites during early pregnancy in mice, and its down-regulation inhibits decidualization of mouse endometrial stromal cells

**DOI:** 10.1186/s12958-015-0047-7

**Published:** 2015-05-27

**Authors:** Yan Gu, Xuan Zhang, Qian Yang, Jian-mei Wang, Ya-ping He, Zhao-gui Sun, Hui-qin Zhang, Jian Wang

**Affiliations:** Shanghai Medical School, Fudan University, Shanghai, China; NPFPC Key Laboratory of Contraceptive Drugs & Devices, Shanghai Institute of Planned Parenthood Research, Shanghai, China; The Second Hospital of Tianjin Medical University, Tianjin, China

**Keywords:** NDRG2, Embryo implantation, Decidualization

## Abstract

**Background:**

N-myc down-regulated gene 2 (NDRG2) is a tumor suppressor involved in cell proliferation and differentiation. The aim of this study was to determine the uterine expression pattern of this gene during early pregnancy in mice.

**Methods:**

Uterine *NDRG2* mRNA and protein expression levels were determined by RT-PCR and Western blot analyses, respectively, during the peri-implantation period in mice. Immunohistochemical (IHC) analysis was performed to examine the spatial localization of NDRG2 expression in mouse uterine tissues. The *in vitro* decidualization model of mouse endometrial stromal cells (ESCs) was used to evaluate decidualization of ESCs following NDRG2 knock down by small interfering RNA (siRNA). Statistical significance was analyzed by one-way ANOVA using SPSS 19.0 software.

**Results:**

Uterine NDRG2 gene expression was significantly up-regulated and was predominantly localized to the secondary decidual zone on days 5 and 8 of pregnancy in mice. Its increased expression was associated with artificial decidualization as well as the activation of delayed implantation. Furthermore, uterine NDRG2 expression was induced by estrogen and progesterone treatments. The *in vitro* decidualization of mouse ESCs was accompanied by up-regulation of NDRG2 expression, and knock down of its expression in these cells by siRNA inhibited the decidualization process.

**Conclusions:**

These results suggest that NDRG2 might play an important role in the process of decidualization during early pregnancy.

## Background

Successful embryo implantation is a critical step in the establishment of pregnancy. Failure of implantation can lead to placental dysfunction, resulting in fetal retardation, preeclampsia (PE), and recurrent miscarriages [1, 2]. A series of complex physiological events occur during the exquisitely regulated process of implantation, including blastocyst migration, apposition and adhesion to the luminal epithelium, extensive degradation and remodeling of the extracellular matrix, and invasion of trophoblast cells into the maternal endometrium, as well as decidualization of endometrial stromal cells (ESCs) [3, 4]. Decidualization, characterized by proliferation and differentiation of ESCs, is a postovulatory process of endometrial remodeling that occurs in preparation for pregnancy. In addition, during pregnancy, the maternal decidua is essential for the establishment and maintenance of immuno-tolerance at the feto-maternal interface, and it regulates placental function and development of the conceptus [5, 6]. However, the exact molecular mechanism underlying decidualization is still unclear. The identification of the factors involved in decidualization would undoubtedly contribute to a better understanding of the implantation process.

N-myc, which belongs to the Myc family, plays an essential role in cell proliferation and differentiation. Although N-myc has been well recognized for its oncogenic bio-function, it has been recently reported to exhibit a dynamic expression pattern during mouse embryonic development [7]. N-Myc downstream-regulated gene 2 (NDRG2), a member of the N-Myc downstream-regulated gene family, encodes a 357-amino acid protein with an apparent molecular weight of 41 kDa [8]. Consistent with its role in cellular differentiation and the stress response, NDRG2 has been identified as a tumor suppressor. Specifically, its expression has been shown to be decreased in a number of different cancer tissues, such as breast [9], liver [10] and gastric cancer tissues [11]. Its expression has also been detected in mouse embryos of various gestational ages. Further, during mouse fetal development, NDRG2 has been found to be strongly expressed in various tissues and organs [12], suggesting its potential role in embryonic development. This gene is phosphorylated by protein kinase B (PKB/Akt), PKC [13], and glucocorticoid-induced kinase 1 (SGK1) [14]. Akt/PKC signaling is a key regulator of trophoblast function during implantation as well as during early placentation [15]. Thus, we hypothesized that NDRG2 might be involved in the process of embryo implantation, and the present study was undertaken to examine the uterine expression pattern of this gene during the estrous cycle and peri-implantation period in mice.

## Methods

### Animals and tissue preparation

Adult ICR mice aged 8-10 weeks were obtained from the SIPPR/BK Laboratory Animal Company (Shanghai, China) and were caged at a controlled temperature (22 °C) under a 14 h light: 10 h dark photoperiod. All experiments were conducted in full compliance with standard laboratory animal care protocols that were approved by the Institutional Animal Care Committee of Shanghai Institute of Planned Parenthood Research (Approval: 2013-19). The estrous cycle was staged by examining vaginal smears as previously described [16], and subsequently the uterine horns were removed from adult females at the diestrus, proestrus, estrous, and metestrus stages (n = 3, per stage) immediately after they were sacrificed by cervical dislocation.

To observe the effects of ovarian steroid hormones on uterine NDRG2 expression, adult females were ovariectomized and allowed to rest for 2 weeks. Then, the ovariectomized mice were randomly divided into four groups (n = 3, per group) and injected with (1) sesame oil (0.1 ml/mouse), (2) 17β-estradiol (E_2_, 100 ng/25 g body weight, Sigma, St. Louis, MO), (3) progesterone (P_4_, 1 mg/25 g body weight, Sigma), or (4) E_2_ (100 ng/25 g body weight) plus P_4_ (1 mg/25 g body weight) according to previously described methods [17–19]. Steroids were dissolved in sesame oil and injected subcutaneously at the same volume (0.1 ml/mouse). The mice were sacrificed at 24 h after treatment, and the uterine horns were collected.

Adult female mice were mated with fertile males of the same strain to achieve pregnancy (day 1 = day of vaginal plug). Pregnancy was confirmed on days 1 and 4 by recovering embryos from the reproductive tracts. Trypan blue dye solution (0.1 % in saline (w/v), 0.1 ml per mouse, Sigma) was injected via the tail vein on day 5 to visualize the implantation sites. The entire uterine horn was collected from the pregnant mice on days 1 and 4 of pregnancy (n = 3, per day). Uterine tissues at the implantation sites (IS) and non-implantation sites (NI) were separately collected from the pregnant mice on days 5 to 8 of pregnancy (n = 3, per day).

Pseudopregnant mice were obtained by mating adult females with vasectomized adult males. Artificial decidualization was induced by intraluminally infusing 25 μl of sesame oil into one uterine horn on day 4 of pseudopregnancy (n = 3), and the contralateral un-injected horn served as a control (n = 3). The mice were sacrificed at 72 h after decidualization was artificially induced [20]. Decidualization was confirmed by both weighing the uterine horns and histological examination of the uterine sections.

In the delayed embryo implantation model, pregnant female mice were bilaterally ovariectomized under ether anesthesia at 08:30–09:00 h on day 4 of pregnancy. The animals in the delayed embryo implantation group and activation group were subcutaneously injected with P_4_ (1 mg/25 g body weight) dissolved in corn oil at 10:00 h from days 4 through 7 of pregnancy to maintain delayed implantation. Then, the animals in the activated implantation group (n = 3) received E_2_ (25 ng/25 g body weight) along with P_4_ to activate embryo implantation [20, 21]. The female mice were euthanized at 10:00 h on day 8 of pregnancy, and the embryos were verified as previously described [22]. Delayed or activated implantation was confirmed by microscopic observation of the metabolically dormant or activated blastocyst in the uterine flush, as previously described [20]. Progesterone keeps the blastocyst dormant so that the inner cellular mass (ICM) remains underdeveloped. However, when estradiol is supplied with progesterone, the blastocyst becomes active, and the ICM becomes more prominent and distinct in appearance [20, 23, 24]. The uterine tissues were collected and then processed for RNA extraction and immunohistochemistry.

### Primary culture of endometrial stromal cells and induction of decidualization *in vitro*

The isolation and culture of mouse ESCs was performed following previously described methods with minor modifications [25]. In summary, uterine horns were collected from pregnant mice on day 4 and cleaned to remove fat tissues. They were then slit longitudinally and washed thoroughly in Hanks’ balanced salt solution (HBSS, Invitrogen, Carlsbad, CA) containing 100 U/ml penicillin (Invitrogen) and 100 μg/ml streptomycin (Invitrogen). Next, the tissues were placed in HBSS containing 10 mg/ml trypsin (Sigma), 6 mg/ml dispase (Invitrogen), 100 U/ml penicillin and 100 μg/ml streptomycin for 1 h on ice, followed by incubation for 1 h at room temperature and 10 min at 37 °C. Following the digestion steps, the tissues were gently mixed, and the supernatant was discarded to remove the endometrial epithelial clumps. The partially digested tissues were then washed twice in HBSS and placed into HBSS containing 0.15 mg/ml collagenase (Invitrogen) at 37 °C for 30 min. Following digestion and shaking, the contents of the tube were passed through a 70 μm gauze filter (Millipore, Darmstadt, Germany) to eliminate epithelial sheets. The cell pellets were washed twice and added to Dulbecco’s modified Eagle’s Medium-F12 medium (DMEM/F12) containing 10 % charcoal-stripped fetal calf serum (FBS, Invitrogen) and antibiotics at 2 × 10^5^ cells per well in a 6-well cell culture plate (Invitrogen). After incubation for 1 h, unattached cells were removed by several washes with HBSS, and cell culturing was continued after the addition of fresh DMEM/F12 containing 1 % charcoal-stripped FBS, 100 U/ml penicillin, 100 μg/ml streptomycin, 10 nM E_2_, and 1 μM P_4_ to induce decidualization of the ESCs.

### siRNA transfection

*NDRG2*-targeting siRNAs were purchased from Santa Cruz Biotechnology, Inc. (*NDRG2* siRNA (m) sc-40758, and sc-3700 7was used as the irrelevant control siRNA, Santa Cruz, Santa Cruz, CA). Prior to the *in vitro* decidualization of ESCs, *NDRG2* siRNAs and control siRNAs were transfected into cultured ESCs according to the siPORT™ NeoFX™ protocol (Ambion/Life Technologies, Grand Island, NY). Briefly, 4 μl of siPORT NeoFX transfection reagent was mixed with 100 nM of siRNA duplexes to form complexes, and this mixture was then dispersed into each well of a 6-well cell culture plate.

### Immunohistochemistry and histological analyses

Uterine tissues were fixed in freshly prepared 4 % buffered paraformaldehyde in phosphate-buffered saline (PBS) at 4 °C for over 40 h. Then, the tissues were dehydrated in graded alcohol and embedded in paraffin (Leica, Wetzlar, Hessen, Germany). Sections of uteri were processed for immunohistochemical detection. Briefly, the sections (5 μm) were deparaffinized and rehydrated in xylene and a graded series of ethyl alcohol, respectively, and then rinsed in PBS. Antigen retrieval was performed by placing the slides in boiling citric acid buffer (10 mmol/l of citrate sodium and 10 mmol/l of citric acid) for 15 min. The sections were cooled to room temperature and sequentially incubated at room temperature with 3 % hydrogen peroxide (H_2_O_2_) in methanol for 15 min to quench endogenous peroxidases. The sections were then incubated with a rabbit anti-NDRG2 primary antibody (Abcam, Cambridge, UK) overnight at 4 °C. After being washed with PBS, the sections were incubated with a biotin-conjugated donkey anti-rabbit secondary antibody (1:200 in blocking solution, Proteintech Company, Wuhan, China). After another wash in PBS, they were incubated with peroxidase-conjugated streptavidin (1:200 in blocking solution, Proteintech Company) for 2 h. Then, they were stained with DAB (DAB, Zhongshan Corp., Beijing, China) according to the manufacturer’s protocol and counterstained with hematoxylin (Sigma). For the negative controls, 10 % donkey serum was used instead of primary antibody. All the sections were examined and photographed under a microscope (DFC420C, Leica).

For vimentin and cytokeratin detection, cultured ESCs were washed in PBS and fixed with PBS containing 4 % paraformaldehyde for 20 min. After being washed in PBS, the cells were incubated in blocking buffer (0.2 % Triton-X100 and 10 % BSA in PBS) for 1 h. Next, they were incubated with blocking buffer containing an anti-vimentin mouse monoclonal antibody (1:100 dilution, Cell Signaling, Beverly, MA), anti-cytokeratin 8 mouse monoclonal antibody (1:50 dilution, Cell Signaling), or negative control mouse IgG antibody at 4 °C overnight. After another wash in PBS, the cells were incubated in blocking buffer containing a biotin-conjugated donkey anti-rabbit antibody (1:200 in blocking solution, Proteintech Company) for 30 min. Then, the cells were again washed in PBS and incubated with peroxidase-conjugated streptavidin (1:200 in blocking solution, Proteintech Company) for 2 h. Next, the samples were stained with DAB (DAB, Zhongshan Corp.) according to the manufacturer’s protocol. Finally, after being washed and stained with hematoxylin, the cells were observed and photographed under a microscope (DFC420C, Leica).

### Real-time PCR analysis

Total RNA was extracted from uterine tissues or ESCs using TRIzol reagent (Invitrogen) according to the manufacturer’s instructions. Extracted RNA was dissolved in diethylpyrocarbonate (DEPC, Sigma)-treated water, and the RNA concentration and purity were estimated by measuring absorbance at 260 and 280 nm with a NanoDrop 2000 (Thermo Scientific, Waltham, MA). cDNAs was synthesized using M-MLV reverse transcriptase (Promega, Madison, WI), according to the manufacturer’s instructions. Real-time PCR was performed in a 20 μl reaction volume using an ABI 7500 thermal cycler (Applied Biosystems, Foster City, CA). The thermal cycling conditions were 95 °C for 30 s, followed by 40 cycles at 94 °C for 5 s and 60 °C for 30 s. Melt curve analysis and agarose gel electrophoresis were then conducted to monitor the purity of the PCR products. Beta-actin was used as an endogenous control. The following primers were used: *NDRG2*, sense, 5′-AGAACTTCGTGCGGGTTCAT-3′, antisense, 5′-TCGCGACAGAATGTAGGCTC-3′; decidual/trophoblast PRL-related protein *(DTPRP)*, sense, 5′-AAGAATGCCCTTCAGCGAGC-3′, antisense, 5′-AGCTGGTGGGTTTGTGACAT-3′; and beta-actin, sense, 5′-GGCTGTATTCCCCTCCATCG-3′, antisense, 5′-CCAGTTGGTAACAATGCCATGT-3′.

### Western blotting

The collected uterine tissues were quickly frozen in liquid nitrogen and granulated into a fine powder. The tissue powder was homogenized in lysis buffer (Beyotime, China). Then, the tissue lysate was centrifuged, and the supernatant was transferred into a new tube. Cultured ESCs were collected in lysis buffer, and the lysate was centrifuged to collect the supernatant. Protein concentrations were measured by Bradford assay (Bio-Rad, Hercules, CA), and 50 μg of total protein was separated on a 12 % acrylamide gel and then transferred electrophoretically onto nitrocellulose membranes (Millipore). The membranes were incubated overnight at 4 °C with specific primary antibodies against NDRG2 and beta-actin (Santa Cruz), followed by incubation with the appropriate secondary antibodies. The blot was developed using a PhosphaGLO AP Substrate Kit (KPL, Gaithersburg, MD) according to the manufacturer’s protocol. All samples were analyzed by Western blot in triplicate. Band intensities were quantified by densitometry using ImageJ software (U.S. National Institutes of Health, MD).

### Statistical analysis

All values were presented as the mean ± SEM, as determined from at least three independent experiments. Statistical significance was assessed by one-way ANOVA. A *P* < 0.05 was considered statistically significant. Statistical analysis was conducted using SPSS 19.0 software (SPSS Software, Chicago, IL).

## Results

### Uterine NDRG2 expression during the estrous cycle and peri-implantation period

**Fig. 1:**
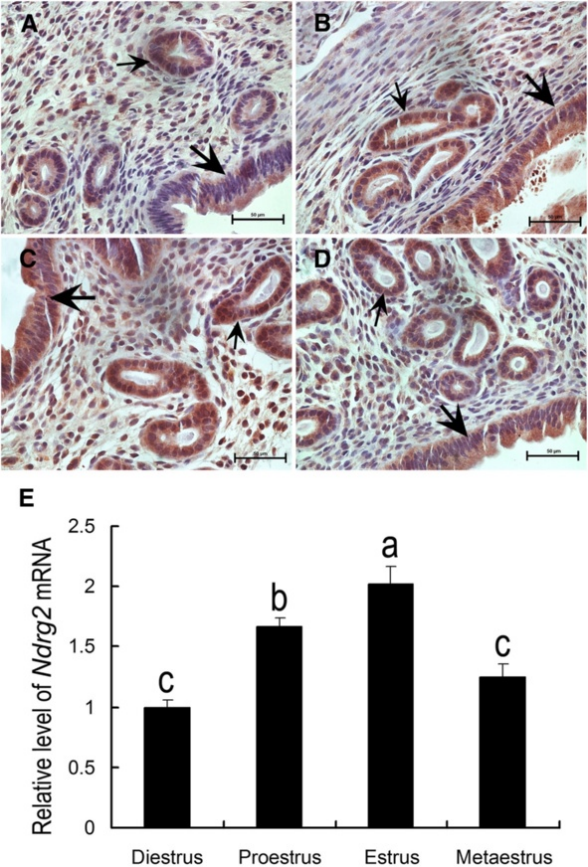
NDRG2 expression in the mouse uterus during the estrous cycle. Upper: immunohistochemical analysis of NDRG2 expression during the diestrus (**a**), proestrus (**b**), estrous (**c**) and metestrus (**d**) phases in the mouse uterus. Below: quantitative PCR analysis of *NDRG2* mRNA expression (**e**) in the uterus during the estrous cycle (n = 3). The thick arrow shows the luminal epithelium. The small arrow indicates the glandular epithelium. The columns with different superscripts are significantly different (*P* < 0.05)

The uterine expression patterns of *NDRG2* mRNA and NDRG2 protein during the mouse estrous cycle were determined by real-time PCR and IHC, respectively. The results showed distinctive NDRG2 protein signals in luminal and glandular epithelial cells at the diestrus (Fig. 1a), proestrus (Fig. 1b), estrous (Fig. 1c) and metestrus phases (Fig. 1d), with the most prominent expression detected during the estrous phase, along with weak positive signals in stromal cells (Fig.1c). Consistently, real-time PCR analysis revealed that *NDRG2* mRNA expression was significantly increased during the estrous phase compared with that during the diestrus, proestrus and metestrus phases (Fig. 1e).

**Fig. 2:**
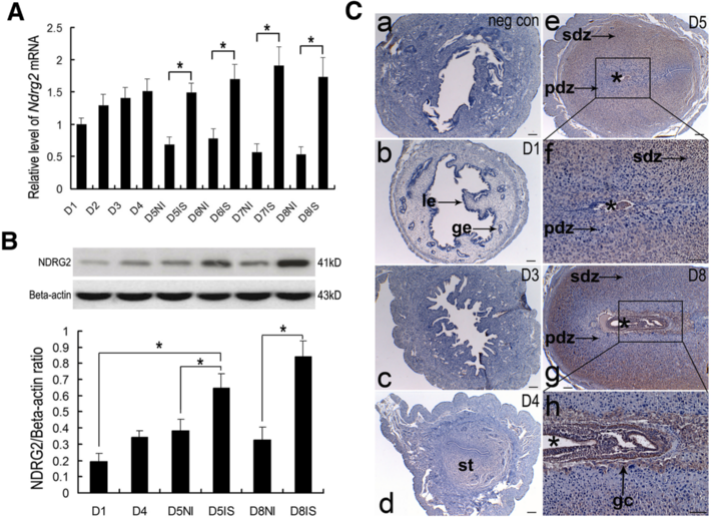
NDRG2 expression in the mouse uterus during early pregnancy. **a** Quantitative PCR analysis of *NDRG2* mRNA expression in the uterus during early pregnancy (n = 3, *P < 0.05). **b** Western blot and densitometric analyses of uterine NDRG2 protein levels during early pregnancy (*n* = 3). All experiments were repeated three times. The data are shown as the mean ± SEM. *P < 0.05. **c** Immunohistochemical analysis of uterine NDRG2 protein expression on days 1, 3, 4, 5, and 8 of pregnancy. IS, implantation sites; NI, non-implantation sites. *, indicates the location of the embryo. le, luminal epithelium; ge, glandular epithelium; st, stroma; pdz, primary decidual zone; sdz, secondary decidual zone; D, day of pregnancy; gc, giant cell; Scale bar represents 100 μm

Prior to the initiation of implantation, uterine *NDRG2* mRNA expression gradually increased from day 1 (D1) to day 4 (D4) of pregnancy. During implantation (day 5 to day 8 of pregnancy), *NDRG2* mRNA expression was differentially regulated between the implantation sites and non-implantation sites. Its expression was significantly up-regulated at the implantation sites, whereas it was obviously down-regulated at the non-implantation sites (Fig. 2a). Meanwhile, the results of Western blot analysis showed that uterine NDRG2 protein expression was significantly increased after the initiation of implantation (day 5 of pregnancy), and its expression at the implantation sites was remarkably higher than that at the non-implantation sites (Fig. 2b). Furthermore, the spatiotemporal expression of NDRG2 protein in the mouse uterus during early pregnancy was examined by IHC. There was no detectable NDRG2 protein signal in the uterine tissue obtained from pregnant mice on day 1 (Fig. 2c/b) or day 3 (Fig. 2c/c). On day 4 of pregnancy, faint NDRG2 protein signals were noted in the subepithelial stromal bed (Fig. 2c/d). On day 5 of pregnancy, the distinct accumulation of NDRG2 protein signals was observed in both the secondary decidual zone and the embryo, with faint expression in the primary decidua immediately adjacent to the implanting embryo (Fig. 2c/e, f). The distribution of NDRG2 protein signals on day 8 of pregnancy was similar to that on day 5, except that the positive signals also accumulated in trophoblasts at implantation sites (Fig. 2c/g, h).

### The effects of steroid hormones on NDRG2 expression in the uterus

**Fig. 3:**
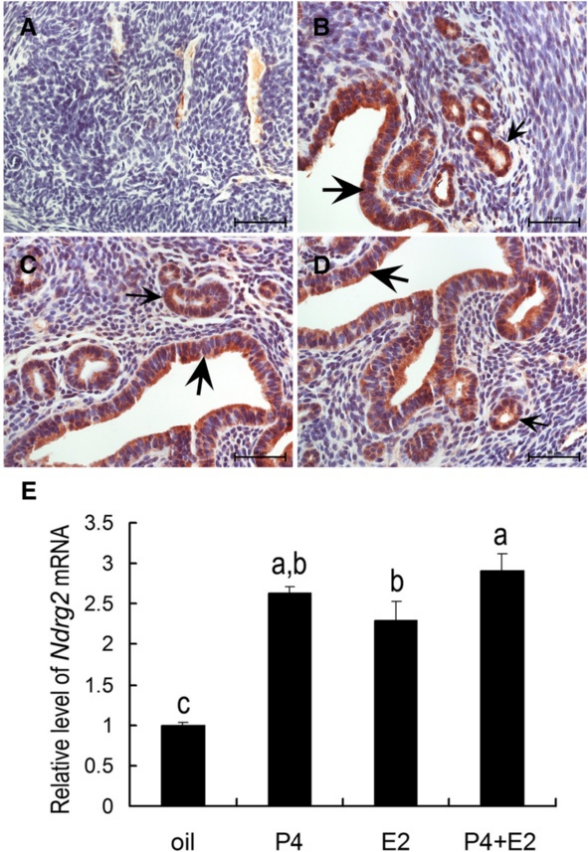
The effects of steroid hormones on NDRG2 expression in the uterus. Upper: immunohistochemical analysis of NDRG2 protein expression in the uteri of ovariectomized mice after oil (**a**), progesterone (**b**), estrogen (**c**), and progesterone plus estrogen treatments (**d**). Below: quantitative PCR analysis of *NDRG2* mRNA expression (**e**) in the uterus following steroid hormone treatment (*n* = 3). The thick arrow indicates the luminal epithelium. The small arrow shows the glandular epithelium. The columns with different superscripts are significantly different (*P* < 0.05)

Ovariectomized mice were used to examine the effects of steroid hormones on NDRG2 expression in the uterus. Compared with the control (Fig. 3a), treatment with E_2_ (Fig. 3b) or P_4_ (Fig. 3c) significantly induced NDRG2 protein expression in both the luminal and glandular epithelia, and the combined administration of E_2_ and P_4_ resulted in a similar effect on its expression (Fig. 3d). A similar alteration in *NDRG2* mRNA expression was observed following treatment with E_2_ or P_4_ (Fig. 3e).

### The association of uterine NDRG2 expression and artificial decidualization and activated implantation

**Fig. 4:**
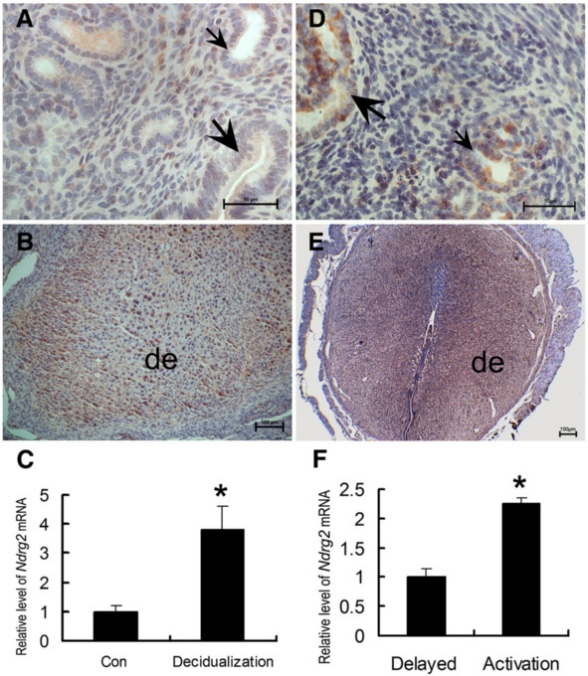
Uterine NDRG2 expression following artificial decidualization and activation of delayed implantation. Immunohistochemical analysis of uterine NDRG2 protein expression following artificial decidualization (**b**) and in its contralateral uninjected uterine horn (**a**), under delayed implantation (**d**) and activation (**e**). Quantitative PCR analysis of *NDRG2* mRNA expression in the uterus following artificial decidualization (**c**) and activation of delayed implantation (**f**) (*n* = 3). The thick arrow indicates the luminal epithelium. The small arrow shows the glandular epithelium. De, decidua; *, significantly different from control (*P* < 0.05)

The artificial decidualization model was used to examine whether uterine NDRG2 expression is dependent on the presence of a living embryo or whether it is induced by the decidualization reaction alone. The results of IHC analysis showed strong NDRG2 protein signals in decidualized stromal cells (Fig. 4b), whereas no visible signals were found in control uteri (Fig. 4a). Meanwhile, uterine *NDRG2* mRNA expression was also found to be significantly up-regulated by artificial decidualization (Fig. 4c). In the delayed implantation model, the NDRG2 protein was abundantly expressed in activated implantation uteri (Fig. 4e), and it was weakly expressed in delayed implantation uteri (Fig. 4d). Likewise, significantly higher *NDRG2* mRNA levels were detected in the activated implantation uteri compared with those in the delayed uteri (Fig. 4f).

### NDRG2 expression in cultured ESCs during the process of *in vitro* decidualization

**Fig. 5:**
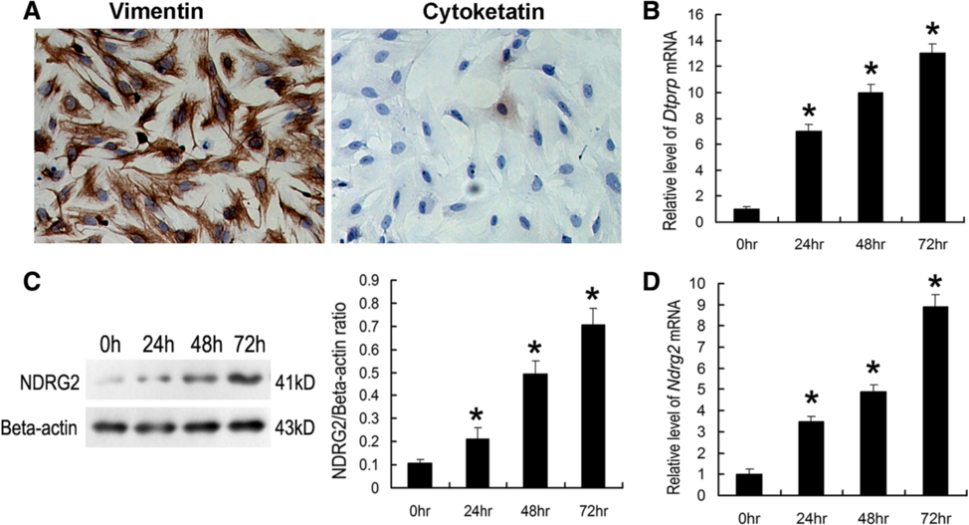
*In vitro* decidualization of mouse primary ESCs. ESCs isolated from the uteri of day 4 pregnant mice were cultured in the presence of P_4_ and E_2_. **a** Immunocytochemical detection of vimentin and cytokeratin in cultured ESCs. **b** Quantitative PCR analysis of *DTPRP* mRNA expression in ESCs cultured for up to 72 h. **c** Western blot analysis of NDRG2 protein expression in ESCs cultured for up to 72 h. Densitometric analyses of NDRG2 at each time point compared with 0 h is shown. **d** Quantitative PCR analysis of *NDRG2* mRNA expression in cultured ESCs. The relative fold induction of *NDRG2* mRNA expression at each time point compared with its expression in the 0 h sample is shown. The values represent the mean ± SEM, as determined from three separate experiments. *, significantly different (*P* < 0.05)

To explore the function of NDRG2 in the decidualization of mouse ESCs, a mouse primary ESCs culture system was established. Positive staining for vimentin and negative staining for cytokeratin were considered to be indicative of ESC purity (Fig. 5a). The transformation of decidual cells from ESCs was indicated by *DTPRP* mRNA expression [26] (Fig. 5b). The mRNA and protein levels of NDRG2 were found to be significantly increased during *in vitro* decidualization (Fig. 5c, d).

### The effects of decreased NDRG2 expression in ESCs on *in vitro* decidualization

**Fig. 6:**
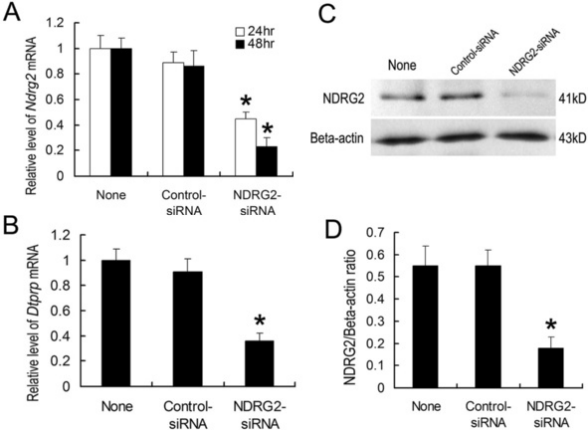
Down-regulation of NDRG2 expression in ESCs inhibits *in vitro* decidualization. Cultured mouse ESCs were transfected with *NDRG2*-targeting siRNAs (100 nM) (a non-targeting siRNA was used as a control) at the time of plating. Quantitative PCR analyses of *NDRG2* mRNA expression (**a**) and *DTPRP* mRNA expression (**b**) in ESCs transfected with *NDRG2*-targeting siRNAs or non-targeting siRNAs at 24 h and 48 h. Western blot (**c**) and densitometric analyses (**d**) of NDRG2 protein levels in ESCs at 72 h after transfection. The relative fold induction of NDRG2 protein expression compared with its expression in non-siRNA-treated group is shown. The values represent the mean ± SEM, as determined from three separate experiments. *, significantly different (*P* < 0.05)

To further explore the role of NDRG2 in the decidualization of ESCs, NDRG2 expression was knocked down in cultured ESCs using targeting siRNAs. As a result, its expression was reduced by more than 60 % in *NDRG2* siRNA-transfected ESCs compared with that in control ESCs (Fig. 6a, c, d), and this reduction was correlated with a significant decrease in *DTPRP* mRNA expression (Fig. 6b), indicating that down-regulation of NDRG2 expression inhibited the decidualization of mouse ESCs.

## Discussion

The present study demonstrated the expression pattern of NDRG2 in the uterus during the estrous cycle and early pregnancy in mice. Uterine NDRG2 expression was found to peak during the estrous phase and to be induced by the ovarian steroid hormones E_2_ and P_4_. During early pregnancy, uterine NDRG2 expression was significantly increased at implantation sites, with predominant localization in the decidual zone. This increase in expression was accompanied by the activation of delayed implantation, as well as artificially induced decidualization, both *in vivo* and *in vitro*. Furthermore, down-regulation of NDRG2 expression in mouse ESCs significantly inhibited decidualization *in vitro*.

The synergistic actions of E_2_ and P_4_ are critical for regulation of the estrous cycle and establishment of uterine receptivity [27, 28]. A putative estrogen-response element (ERE) is located in the promoter region of the *NDRG2* gene, and NDRG2 expression in astrocytes is regulated by estrogen [29, 30]. In the present study, we found that both E_2_ and P_4_ had stimulatory effects on uterine NDRG2 expression in ovariectomized mice (Fig. 3). In addition, during the normal estrous cycle, uterine NDRG2 expression was significantly increased during the proestrus and estrous phases, whereas it was decreased during the metestrus and diestrus phases (Fig. 1), consistent with the E_2_ surge that takes place during the proestrus and estrous phases [31]. Because NDRG2 expression was detected in luminal and glandular epithelial cells as well as in stromal cells, we speculated that it might participate in regulating cyclic proliferation and differentiation in endometrial epithelial/stromal cells to optimally prepare for embryo implantation under the control of E_2_ and P_4_ during the estrous cycle.

Successful embryo implantation is integral to the establishment of pregnancy, and initiation of implantation coincides with the establishment of uterine receptivity and the subsequent decidualization of endometrial stromal cells (ESCs) [32]. In the mouse, a pre-ovulatory E_2_ surge stimulates uterine epithelial cell proliferation on day 1 of pregnancy, followed by a rise in the P_4_ level and its secretion by the newly formed corpora lutea, which initiates uterine stromal cell proliferation on day 3. Embryos enter the uterus at midnight on day 3 or in the early hours of day 4 after the uterus is exposed to an increased concentration of P_4_ for at least 24 h on day 3 followed by exposure to estrogen, causing it to be receptive for embryo implantation [33]. We found that uterine NDRG2 expression remained increased during the pre-implantation period from day 1 to day 4, suggesting its potential role in establishing uterine receptivity under the regulation of ovarian steroids.

Once mouse embryos attach to a receptive endometrium, decidualization is triggered by extensive proliferation and differentiation of ESCs into decidual stromal cells (DSCs) at embryo implantation sites. Because the decidual response can be induced in a reproducible manner in the absence of an embryo and decidual zones are easily discernable, the mouse is a good model to investigate the mechanism of decidualization. In the mouse, at least three factors may be necessary for normal decidualization, including E_2_, P_4_ and embryonic or physical stimulation (by intra-luminal infusion of oil or scratching with a needle). Increases in E_2_ and P_4_ stimulate the proliferation and differentiation of ESCs surrounding invading trophoblast cells to support the decidualization process [33–35]. In the present study, a significant increase in NDRG2 expression was observed at implantation sites on days 5 and 8 of pregnancy in the mice, and NDRG2 protein signals were predominantly localized to the decidual zone. Moreover, because E_2_ and P_4_ induced NDRG2 expression in the uterus and increased its expression in ESCs following artificial decidualization (Fig. 4 and Fig. 5), we hypothesized that it might be involved in the decidualization of these cells. To further investigate this hypothesis, we specifically knocked down NDRG2 expression in mouse ESCs using *NDRG2*-targeting siRNAs and subsequently evaluated the effect of decreased NDRG2 expression on ESC decidualization *in vitro* using DTPRP as a marker of decidualization. Decreased expression of this gene was found to remarkably inhibit decidualization *in vitro* (Fig. 6), indicating that it may participate in this process.

An implantation site is distinguished from a non-implantation site not only by the presence of DSCs and trophoblast cells but also by the enrichment of decidual immune cells. Dendritic cells (DCs) are the most predominant immune cells in decidua at implantation sites, and they play a critical role in preserving a hospitable micro-environment for pregnancy [36]. Although NDRG2 expression in decidual DCs has not been reported, its expression in DCs derived from CD^34+^ progenitor cells has been detected. Inhibition of DCs differentiation is accompanied by reduced NDRG2 expression, and the down-regulation of its expression negatively affects the ability of DCs to stimulate T cells proliferation [37]. Thus, an increased in NDRG2 expression at implantation sites during early pregnancy might also be associated with the differentiation and maturation of decidual DCs. NDRG2 expression in decidual DCs will be explored further by our group.

Although the exact role of NDRG2 in regulating ESCs and DSCs needs to be further explored, NDRG2 has been reported to contain several potential phosphorylation sites and to be phosphorylated by Akt [13] and SGK1 [14]. Importantly, a decrease in uterine Akt expression has been shown to result in abnormal decidualization in mice [38], and reduced SGK1 expression in DSCs has been observed in recurrent spontaneous miscarriage (RSM) patients [39]. Thus, it is reasonable to believe that the function of NDRG2 in ESCs and DSCs might be partially regulated by the Akt or/and SGK1 signaling pathway(s). Conversely, the appropriate invasion of trophoblast cells into maternal stroma is also a pivotal event that occurs during implantation [40]. Because we observed NDRG2 protein expression in trophoblast cells at implantation sites on day 8 of pregnancy in the mice in this study (Fig. 2) and it has been demonstrated that E_2_ and P_4_ participate in the regulation of trophoblast cells [41, 42], we hypothesize that NDRG2 might also be involved in the invasion of trophoblast cells during early pregnancy.

## Conclusions

In summary, the present study has demonstrated that steroid hormones stimulate NDRG2 expression in the uterus and that NDRG2 expression is significantly up-regulated at implantation sites during early pregnancy in mice. During *in vivo* and *in vitro* artificial decidualization, NDRG2 expression was found to be remarkably increased. Further, down-regulation of its expression in ESCs inhibited *in vitro* decidualization. These results suggest that NDRG2 might be essential for embryo implantation and decidualization.

## Abbreviations

DCs: Dendritic cells
DEPC: Diethylpyrocarbonate
DRPRP: Decidual/trophoblast PRL-related protein
DSCs: Decidual stromal cells
ESCs: Endometrial stromal cells
ERE: Estrogen response element
E_2_: 17β-estradiol
FBS: Fetal calf serum
HBSS: Hanks’ balanced salt solution
H_2_O_2_: Hydrogen peroxide
IHC: Immunohistochemistry
NDRG2: N-myc down-regulated gene 2
PBS: Phosphate-buffered saline
PE: Preeclampsia
PKB: Protein kinase B
P_4_: Progesterone
RSM: Recurrent spontaneous miscarriage
siRNA: small interfering RNA
SGK1: Glucocorticoid-induced kinase 1
