# Erratum: Uterine NDRG2 expression is increased at implantation sites during early pregnancy in mice, and its down-regulation inhibits decidualization of mouse endometrial stromal cells

**DOI:** 10.1186/s12958-015-0089-x

**Published:** 2015-08-29

**Authors:** Yan Gu, Xuan Zhang, Qian Yang, Jian-mei Wang, Ya-ping He, Zhao-gui Sun, Hui-qin Zhang, Jian Wang

**Affiliations:** Shanghai Medical School, Fudan University, Shanghai, China; NPFPC Key Laboratory of Contraceptive Drugs & Devices, Shanghai Institute of Planned Parenthood Research, Shanghai, China; The Second Hospital of Tianjin Medical University, Tianjin, China

After publication of this article [1], the authors noticed an error in Fig. 5: in panel c, the bands produced by NDRG2 and beta-actin were incorrectly labelled. The original version of this article has been updated to correct this. The correct figure is shown below:

**Fig. 5:**
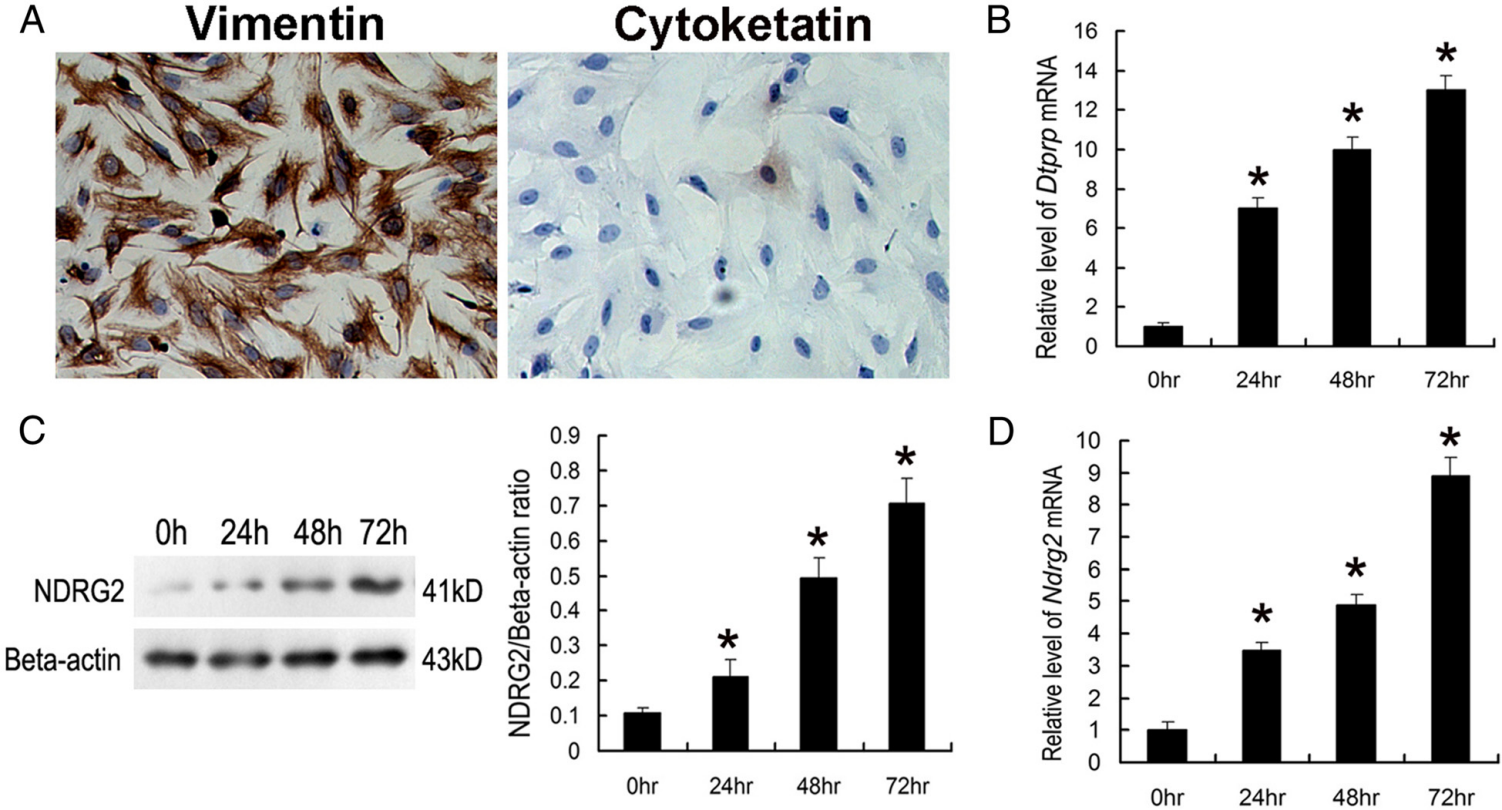
In vitro decidualization of mouse primary ESCs. ESCs isolated from the uteri of day 4 pregnant mice were cultured in the presence of P4 and E2. **a** Immunocytochemical detection of vimentin and cytokeratin in cultured ESCs. **b** Quantitative PCR analysis of DTPRP mRNA expression in ESCs cultured for up to 72 h. **c** Western blot analysis of NDRG2 protein expression in ESCs cultured for up to 72 h. Densitometric analyses of NDRG2 at each time point compared with 0 h is shown. **d** Quantitative PCR analysis of NDRG2 mRNA expression in cultured ESCs. The relative fold induction of NDRG2 mRNA expression at each time point compared with its expression in the 0 h sample is shown. The values represent the mean ± SEM, as determined from three separate experiments. *, significantly different (P < 0.05)
